# Small RNA and Degradome Deep Sequencing Reveals the Roles of microRNAs in Seed Expansion in Peanut (*Arachis hypogaea* L.)

**DOI:** 10.3389/fpls.2018.00349

**Published:** 2018-03-20

**Authors:** Xingli Ma, Xingguo Zhang, Kunkun Zhao, Fapeng Li, Ke Li, Longlong Ning, Jialin He, Zeyu Xin, Dongmei Yin

**Affiliations:** College of Agronomy, Henan Agricultural University, Zhengzhou, China

**Keywords:** peanut, deep sequencing, miRNA, degradome, seed expansion

## Abstract

Seed expansion in peanut is a complex biological process involving many gene regulatory pathways. MicroRNAs (miRNAs) play important regulatory roles in plant growth and development, but little is known about their functions during seed expansion, or how they contribute to seed expansion in different peanut lines. We examined seed miRNA expression patterns at 15 and 35 days after flowering (DAF) in two peanut eighth-generation recombinant inbred lines (RIL8); 8106, a medium-pod variety, and 8107, a super-pod variety. Using high-throughput sequencing, we identified 1,082 miRNAs in developing peanut seeds including 434 novel miRNAs. We identified 316 differentially expressed miRNAs by comparing expression levels between the two peanut lines. Interestingly, 24 miRNAs showed contrasting patterns of expression in the two RILs, and 149 miRNAs were expressed predominantly in only one RIL at 35 DAF. Also, potential target genes for some conserved and novel miRNAs were identified by degradome sequencing; target genes were predicted to be involved in auxin mediated signaling pathways and cell division. We validated the expression patterns of some representative miRNAs and 12 target genes by qPCR, and found negative correlations between the expression level of miRNAs and their targets. miR156e, miR159b, miR160a, miR164a, miR166b, miR168a, miR171n, miR172c-5p, and miR319d and their corresponding target genes may play key roles in seed expansion in peanut. The results of our study also provide novel insights into the dynamic changes in miRNAs that occur during peanut seed development, and increase our understanding of miRNA function in seed expansion.

## Introduction

As one of the most important oil crops in the world, peanut (*Arachis hypogaea* L.) is grown on six continents but mainly in Asia, Africa, and America (Zhang et al., 2012; Zhao et al., 2015). Yield of peanut is determined primarily by two vital factors; (1) seed expansion, and (2) subsequent seed filling (Stanciel et al., 2000; Ramakrishna et al., 2006). However, compared to other food crops, peanuts show a distinct pattern of seed development. After fertilization, a new organ called the peg differentiates from the ovary (Brennan, 1969). Little mitotic division occurs in the embryo or endosperm during active geotropic peg growth until the peg penetrates the soil. Rapid embryo cell division then begins a few days later. This occurs approximately 10–12 days following fertilization (Smith, 1956). Seed developmental changes in water content, nucleic acid levels, enzyme activities, and storage-protein deposition patterns were studied from the earliest stage when kernels can be removed (Aldana et al., 1972; Basha and Cherry, 1976; Yamada et al., 1980). It has been shown that developmentally expressed genes in seeds are regulated at the level of transcription initiation (Falvey and Schibler, 1991). In addition, some important genes that contribute highly to seed development have also been identified in peanut (Bi et al., 2010; Zhang et al., 2012). Although some seed development genes have been studied in peanut, the roles of the upstream regulators of these coding genes, such as miRNAs, have not been well studied.

MicroRNAs (miRNAs) are a class of small (19–24 nt), non-coding RNAs that can regulate gene expression by cleavage or translational repression of the target gene mRNAs (Van Ex et al., 2011). miRNAs have been shown to play important roles in plant growth, development, and the response to environmental stresses (Sunkar and Zhu, 2004; Ding et al., 2012; Feng et al., 2014; Zhao et al., 2016). For example, in *Arabidopsis*, 33 different miRNA families have been detected in mature pollen, and several (miR156, miR2939, miR158, and miR845) showed elevated expression levels in pollen compared with leaves (Grant-Downton et al., 2009). In rice, miR167 may play a role in rice grain filling through the auxin-miR167-ARF8-OsGH3.2 regulatory pathway (Xue et al., 2009); miR397 is highly expressed in young panicles and grains (Zhang et al., 2013). In peanut, studies on miRNAs also have been reported (Zhao et al., 2010; Chi et al., 2011), and several miRNAs (miR160, miR164, miR393, miR396, miR397, miR482, and miR2118) have possible roles in the response to pathogen infection (Zhao et al., 2015). Although some miRNAs have been identified in peanut, to date, the mechanism of seed expansion involving miRNAs is unclear.

To understand the role of miRNAs in peanut seed expansion, we used two representative peanut lines in this study; 8106 and 8107 are eighth-generation recombinant inbred lines (RIL8) from a cross between cultivars of Huayou7 (female) and Huayou4 (male), both of which are erect Virginia-types with 8–10 branches. The main difference between the two RILs is the pod size: line 8106 has medium-sized pods (3.2 cm long × 1.3 cm wide), and a 100-seed weight of 100 g, while line 8107 has super-large pods (5.5 cm × 2.07 cm) with a corresponding 100-seed weight of 182 g. These two typical peanut lines were investigated to help us understand peanut seed expansion by analyzing seed miRNA expression patterns and their target genes.

## Materials and Methods

### Plant Materials

**FIGURE 1:**
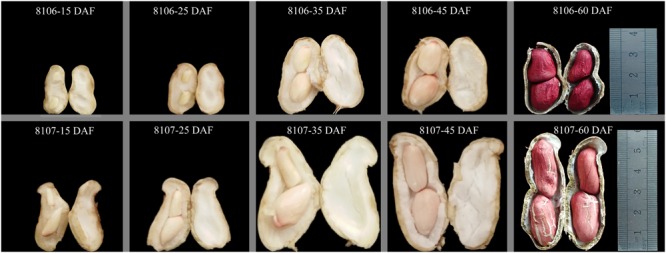
Morphological changes in two peanut lines during seed development. 8106 and 8107 lines indicate the two peanut recombinant inbred lines (RILs). 15 DAF, 25 DAF, 35 DAF, 45 DAF, and 60 DAF indicate 15, 25, 35, 45, and 60 days after flowering, respectively.

Seeds of the two peanut lines were sown on 15 May 2016 in the field at Henan Agricultural University (Zhengzhou, China; E113°41′, N34°49′, 94 m altitude), where the average temperature is 14.4°C and the average rainfall is 632 mm per year. Three replicated plots of both peanut lines were planted in the field; row length was 6 m, the inter-row spacing was 40 cm, and the within-row spacing was 20 cm. Peanut pod development can be divided into two periods: pod expansion and pod filling. Generally, the first sign of pod development was seen at 15 days after flowering (DAF), and the pods enlarge to their maximum size at about 35 DAF, which was named the stereotyped fruit. The peanut pods mature at about 60 DAF (**Figure 1**). In our study, we selected seeds at 15 and 35 DAF in which to study seed expansion in peanut. Fresh seeds were harvested from plants of both peanut RILs at 15 DAF and 35 DAF. Four samples were frozen immediately in liquid nitrogen and stored at -80°C until use. Three replicates of samples were collected in this study.

### Small RNA Library Construction and DNA Sequencing

Total RNA was extracted using TRK-1001 following the manufacturer’s instructions. RNA quantity and purity were assessed using a Bioanalyzer 2100 and the RNA 6000 Nano LabChip Kit (Agilent Technologies, Santa Clara, CA, United States) with RIN number > 7.0. Total RNA was ligated to the RNA 3′ and RNA 5′ adapters and reverse transcription, followed by PCR, was performed to make cDNA constructs of the small RNAs. The small cDNA fractions that ranged from 22 to 30 nt in length were then isolated via 6% denaturing polyacrylamide gel electrophoresis. Finally, the cDNA constructs were purified, and the library was validated. We then performed single-end sequencing (50 bp) on an Illumina Hiseq2500 at the LC-BIO (Hangzhou, China) following the vendor’s recommended protocol.

### Identification of Known and Potential Novel miRNAs

The raw sequencing reads were subjected to the Illumina pipeline filter (Solexa 0.3), and the dataset was then further processed using an in-house program, ACGT101-miR (LC Sciences, Houston, TX, United States) to remove adapter dimers, low complexity reads, common RNA families (rRNA, tRNA, snRNA, snoRNA), repeats in the NCBI GenBank^1^, Repbase^2^, and Rfam^3^) databases. Subsequently, the remaining non-annotated sequences were mapped to specific species precursors in miRBase 21.0^4^ by BLAST searches to identify known miRNAs. On the basis of sequence similarity to peanut genome, these miRNA sequences were classified into subgroups 1a, 1b, 2a, 2b, and 3 with high-to-mid confidence in order (Ambros et al., 2003; Li et al., 2017). The classification criteria for each subgroup were as follows: in gp1a, reads were mapped to miRNAs/pre-miRNAs of specific species in miRbase and the pre-miRNAs were further mapped to genome (known miRNAs); in gp1b, reads were mapped to (except for specific species) miRNAs/pre-miRNAs of selected species in miRbase and the pre-miRNAs were further mapped to genome (conserved miRNAs); in gp2a, reads were mapped to miRNAs/pre-miRNAs of selected species in miRbase and the mapped pre-miRNAs were not further mapped to a genome, but the reads (and of course the miRNAs of the pre-miRNAs) were mapped to a genome. The extended genome sequences from the genomic loci may form hairpins (conserved miRNAs); in gp2b, reads were mapped to miRNAs/pre-miRNAs of selected species in miRbase and the mapped pre-miRNAs were not further mapped to a genome, but the reads (and of course the miRNAs of the pre-miRNAs) were mapped to a genome. The extended genome sequences from the genomic loci may not form hairpins (conserved miRNAs); in gp3, reads were mapped to miRNAs/pre-miRNAs of selected species in miRbase and the mapped pre-miRNAs were not further mapped to a genome, and the reads were also not mapped to a genome (conserved miRNAs). All non-annotated reads with lengths of 18–25 nt were mapped to the reference genome sequences of two diploid ancestors of cultivated peanut, *A. ipaensis*^5^ and *A. duranensis*^6^ using the Bowtie package; only perfectly matched sRNAs were used in further analyses. Novel miRNAs were identified using the MIREAP software^7^ based on their precursors, and the hairpin RNA structures containing sequences were predicated from the flanking 120 nt sequences using RNAfold software^8^. The key criteria for miRNA prediction were based on those that had been previously reported in the literature (Meyers et al., 2008).

### Analysis of Differentially Expressed miRNAs

The raw reads for each small RNA sequence were first normalized using global normalization procedures (Huber et al., 2002), and the differentially expressed sequence counts at 15 and 35 DAF were then analyzed using the online web service IDEG6^9^. Because we included three biological replicates in the sequencing, each miRNA derived from 15 and 35 DAF seeds were compared using the *T*-Test. Only *p*-values <0.05 were considered to be differentially expressed miRNAs.

### Reverse Transcription Reactions

Reverse transcription reactions were performed using the One Step PrimeScript miRNA cDNA Synthesis Kit (TaKaRa Co., Tokyo, Japan) following the manufacturer’s instructions. First-strand cDNA was synthesized in 20-μl reaction volumes containing 1 μg total RNA, 10 μl 2X miRNA reaction buffer mix, 2 μl 0.1% BSA, 2 μl miRNA PrimeScript RT enzyme mix, and RNase-free dH_2_O (to 20 μl). The reactions were incubated at 37°C for 60 min, followed by 85°C for 5 min, and then stored at -20°C until use.

### Validation of Differentially Expressed miRNAs

The qPCR reactions were performed with a SYBR PrimeScript miRNA RT-PCR Kit on a Fluorescence detection system (TianGen Biotech, Beijing, China). Each 20 μl reaction contained 1 μl cDNA template (∼100 ng), 1 μl 10 μM PCR forward primer, 1 μl 10 μM Uni-miR qPCR primer, 10 μl 2× SYBR premix EX TaqII, and 7 μl ddH_2_O. The amplification reactions were performed by first incubating at 95°C for 5 min, followed by 45 cycles of 95°C for 15 s, 60°C for 30 s, and 72°C for 45 s. Following amplification, a threshold was set and the threshold cycle (CT) was recorded automatically. All reactions were performed in triplicate for each sample. The relative expression levels of the miRNAs were calculated using the 2^-ΔΔCT^ method (Livak and Schmittgen, 2001) and the data were normalized to the CT values for the *Actin* gene. The primer sequences for 16 differentially expressed miRNAs are given in **Supplementary Table S13-1**.

### Degradome Library Construction and Target Identification

Total RNA was extracted using TRK1001 following the manufacturer’s instructions. The total RNA quantity and purity were determined using a Bioanalyzer 2100 and RNA 6000 Nano LabChip Kit (Agilent, Santa Clara, CA, United States) with RIN number > 7.0. Approximately 20 μg of total RNA was used to prepare the Degradome library. The method differed considerably from previous efforts (Addo-Quaye et al., 2008, 2009b) and followed the method of (Ma et al., 2010) with some modifications: (1) the poly(A)+ RNA was bound to the mRNA Capture Beads, (2) the 5′ adapter was ligated to only those RNAs containing 5′-monophosphates, (3) reverse transcription was performed using 3′-random primers, (4) the library was PCR amplified, and (5) the libraries were sequenced using the 5′ adapter only.

The purified cDNA library was used for cluster generation on Illumina’s Cluster Station and then sequenced on the Illumina Hiseq 2500 instrument following the manufacturer’s instructions. The extracted sequencing reads were stored in file *SampleA_RawData.txt* and were then used in e standard data analysis, which is described in Mao et al. (2012). A publicly available software package, CleaveLand3.0, was used to analyze the sequencing data generated. The key functions performed by this software and the relevant analysis results are described by Addo-Quaye et al. (2009a).

### Gene Ontology (GO) and KEGG Analysis of Target Genes

Gene ontology (GO) annotations of target genes corresponding to the differentially expressed miRNAs were downloaded from the Gene Ontology^10^, NCBI^11^, and UniProt^12^. The KEGG^13^ pathway was analyzed through the ClueGO plug-in^14^ and Cytoscape software V2.8.2^15^ to identify the significant pathways of the differential genes. GO terms and KEGG pathways were regarded to be significantly enriched with the corrected *P*-value ≤0.05, which was calculated using hypergeometric test (Hou et al., 2017).

### Validation of the Target Genes

Expression analysis of selected target genes was performed by qPCR. First-strand cDNA was synthesized from 1 μg of RNA using TransScript First-Strand cDNA Synthesis SuperMix (TransGen Co., Beijing, China) following the manufacturer’s instructions. Each 20 μl qPCR reaction contained 1 μl cDNA, 0.5 μl 10 μM forward primer, 0.5 μl 10 μM reverse primer, 10 μl 2× TransStart Top Green qPCR SuperMix, and 8 μl double-distilled water. The amplification conditions were an initial denaturation step of 95°C for 5 min, followed by 45 cycles of 95°C for 15 s, 56°C for 30 s, and 72°C for 45 s. All reactions were performed in triplicate for each sample. Relative expression levels of the targets were quantified using the 2^-ΔΔCT^ method (Livak and Schmittgen, 2001) normalized to *Actin* CT values. The sequences of the primer pairs for the target genes are given in **Supplementary Table S13-2**.

## Results

### Sequencing and Annotation of Peanut miRNAs

Lines 8106 and 8107 showed obvious difference in seed size (**Figure 1**). To investigate the dynamic variation of miRNAs during peanut seed expansion, two seed developmental stages, 15 DAF and 35 DAF, were selected to sequence the small RNAs using Solexa high-throughput sequencing technology. From RIL 8106 at the 15 DAF (C1) and 35 DAF (T1), we obtained 17,065,477 and 15,320,325 unfiltered sequence reads, respectively; RIL 8107 at 15 DAF (C2) and 35 DAF (T2) yielded 13,887,551 and 13,804,333 unfiltered sequence reads, respectively, from the miRNA libraries. We obtained 7,145,081, 6,008,924, 5,339,568, and 8,578,303 unique sequences from the C1, T1, C2, and T2 libraries, respectively (**Supplementary Table S1**). After discarding low-quality reads and further filtering the Rfam (rRNA, tRNA, snoRNA, snRNA, and other Rfam RNAs) and Repbase sequences, a total of 6,466,666, 5,121,789, 4,548,767, and 7,786,168 unique valid small RNA sequences remained, respectively (**Supplementary Table S1**). The lengths of the unique valid reads ranged from 18 to 25 nucleotides (nt), and the 21–24 nt sequences were predominant in the C1, T1, C2, and T2 libraries, with the 24 (nt) sequences being the most common (**Supplementary Figure S1**).

### Identification of Known, Conserved, and Novel miRNAs

To identify the known miRNAs at different stages of seed expansion, the valid reads from each dataset were mapped to the precursor sequences in the peanut miRNA database available in miRBase (release 21). Based on the screening criteria of the miRNAs, we identified a total of 72 known miRNAs and 576 conserved miRNAs from the four small RNA libraries (**Supplementary Table S2**). Among these, 35 known miRNAs and 320 conserved miRNAs were shared in common (**Supplementary Figures S2A,B**). A total of 89 known and conserved miRNAs were confirmed in miRbase. In addition, 337 miRNAs were also confirmed, but their sequences were different from those reported in miRbase, including miR156b-5p_R+1, miR167-3p_L-1, miR394_L+1_2, miR408-5p_L+1R-1, and miR3508_2ss20CT21AT, among others. Furthermore, 222 new miRNAs were identified for newly reported 5p or 3p sequences, such as miR3510-p5_1ss22GT, miR3513-p5, miR3515-p3, miR3516-p5, and miR3517-p5 (**Supplementary Table S2**).

To identify the novel or predicted candidate miRNAs (PC miRNAs), the criteria for annotation of plant miRNAs (Meyers et al., 2008) were used in our study. The classification criteria for the PC miRNAs are as follows: reads were not mapped to pre-miRNAs of selected species in miRbase, but the reads were mapped to the genome and the extended genome sequences from the genome loci may form hairpins. We predicted 434 PC miRNAs from the four small libraries (332 miRNAs in C1, 332 miRNAs in T1, 241 miRNAs in C2, and 321 miRNAs in T2) (**Supplementary Table S3**). Among these, 172 PC miRNAs were found to be common to the four miRNA databases (**Supplementary Figure S2C**).

### Comparison of Differentially Expressed miRNAs Between the Two Peanut RILs

**FIGURE 2:**
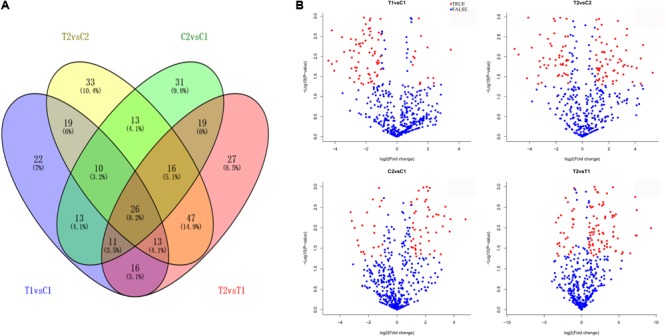
Differentially expressed miRNAs in two peanut RILs. Venn diagrams showing the number of common and specific miRNAs in comparisons of the four libraries **(A)**. Volcanic diagrams showing the number of differentially expressed miRNAs in each comparison **(B)**. The red dots indicate miRNAs with significant differences, and the blue dots indicate that the difference was not significant for miRNA expression. C1, RIL 8106 at 15 DAF; T1, RIL 8106 at 35 DAF; C2, RIL 8107 at 15 DAF; T2, RIL 8107 at 35 DAF.

**FIGURE 3:**
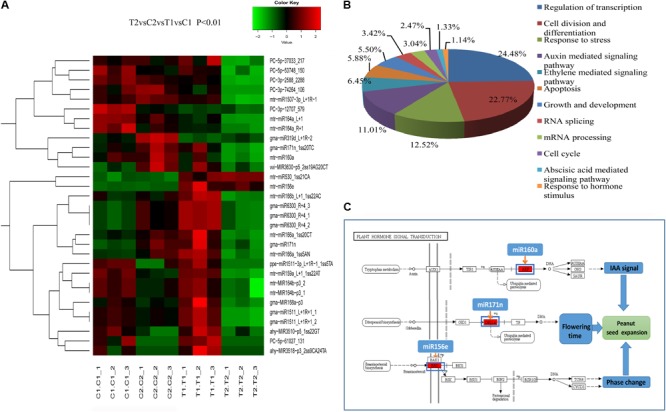
Clustering of partially differentially expressed miRNAs in the two peanut RILs **(A).** Biological Process GO categories of the predicted target genes of the differentially expressed miRNAs **(B)**. KEGG pathways related to plant hormone signal transduction targeted by differentially expressed miRNAs **(C)**.

We compared the frequencies of occurrence of differentially expressed miRNAs at the 15 and 35 DAF stages between the two lines based on a Poisson distribution approach (Audic and Claverie, 1997). We identified 130, 177, 139, and 175 differentially expressed miRNAs in the T1 vs. C1, T2 vs. C2, C2 vs. C1, and T2 vs. T1 comparisons, respectively (**Supplementary Tables S4-1–S4-4**). Among these, 26 differentially expressed miRNAs were common to the four comparisons (**Figure 2**). In order to dissect the miRNAs at the pod enlargement stage, we mainly focused on differentially expressed miRNAs at the 15 DAF and 35 DAF stages between the two peanut RILs. We identified 86 known or conserved miRNAs from RIL 8106 and 119 known or conserved miRNAs from RIL 8107 that were differentially expressed at the 35 DAF stage compared to 15 DAF (**Supplementary Tables S4-1,4-2**). Of these, 48 miRNAs were common to the two peanut lines (**Supplementary Figure S3A**). Moreover, 38 miRNAs and 71 miRNAs were specifically expressed in RILs 8106 and 8107, respectively (**Supplementary Figure S3A**). We used the following criteria to identify differentially expressed miRNAs: the adjusted *p*-value was <0.05 in at least one of the two comparisons. Finally, 143 miRNAs were differentially expressed at 35 DAF in the two lines (**Supplementary Table S5**), and 11 miRNAs showed up-regulated expression (**Table 1**), while another 21 miRNAs were down-regulated (**Table 2**). There are 16 miRNAs showed contrasting expression patterns in the two lines (**Table 3**); among them, 10 miRNAs, such as miR159a-5p_2ss19TA20TA, miR167-3p_L-1, and miR172c-5p, were down-regulated in RIL 8106 but up-regulated in RIL 8107, while six miRNAs including miR166a_1ss5AN, miR166b_L+1_1ss22AC, and miR6300_R+4_1 were down-regulated in RIL 8107 but were up-regulated in RIL 8106 (**Figure 3A**). Moreover, 95 miRNAs were expressed predominantly in only one of the RILs at the 35 DAF stage (**Table 4** and **Supplementary Table S6**).

**Table 1:** miRNAs showing up-regulated expression in two peanut recombinant inbred lines at 35 DAF.

miRNA ID	Normalized value mean				Fold-change	Log_2_	Fold-change	Log_2_	*p*-Value	*p*-Value
	C1	T1	C2	T2	(T1/C1)	(T1/C1)	(T2/C2)	(T2/C2)	(T1 vs. C1)	(T2 vs. C2)
ahy-miR156b-3p	110	312	113	344	2.84	1.51	3.04	1.60	4.35E-02	6.50E-03
ahy-miR156b-5p_R+1	16	32	10	28	2.08	1.06	2.74	1.45	9.20E-03	5.73E-04
ahy-miR156b-5p_R+2	0	18	0	52	inf	inf	inf	inf	4.59E-02	1.96E-02
ahy-miR156e	1,500	7,809	1,535	5,764	5.21	2.38	3.75	1.91	4.53E-02	1.43E-02
ahy-miR167c_L+1_1ss22GT	1,452	2,256	948	1,933	1.55	0.64	2.04	1.03	7.40E-03	2.78E-03
ahy-miR482a-5p_L-1_2ss11TG20AT	27	76	57	1,366	2.79	1.48	24.03	4.59	4.86E-02	4.08E-03
ahy-miR530_1ss21CA	394	736	395	826	1.87	0.90	2.09	1.06	1.27E-02	1.78E-04
ahy-miR3518	112	440	166	323	3.92	1.97	1.94	0.96	5.01E-03	4.14E-02
ahy-miR3518-p5	11	81	29	68	7.19	2.85	2.31	1.21	4.97E-02	3.81E-02
ahy-miR3519	1	9	0	10	11.07	3.47	inf	inf	5.88E-03	3.84E-02
ahy-miR8723b-p3_2ss6AC21TC	14	27	13	32	1.90	0.93	2.47	1.30	4.08E-04	2.18E-03

**Table 2:** Down-regulated miRNAs in two peanut recombinant inbred lines at 35 DAF.

miRNA ID	Normalized value mean				Fold-change	Log_2_	Fold-change	Log_2_	*p*-Value	*p*-Value
	C1	T1	C2	T2	(T1/C1)	(T1/C1)	(T2/C2)	(T2/C2)	(T1 vs. C1)	(T2 vs. C2)
ahy-miR1509b-p5_2ss11AG18GA	1,433	687	1,237	822	0.48	–1.06	0.66	–0.59	1.73E-04	7.90E-04
ahy-miR156o-p3_2ss21GA23TA	44	0	137	50	–inf	–inf	0.36	–1.47	4.34E-04	1.39E-02
ahy-miR160e-p3_1ss5GA	443	131	464	198	0.30	–1.76	0.43	–1.23	2.85E-04	1.53E-02
ahy-miR164a_L+1	104	53	73	12	0.50	–0.99	0.17	–2.58	8.46E-03	1.53E-02
ahy-miR164a_R+1	94	38	52	6	0.41	–1.30	0.11	–3.19	3.04E-02	1.68E-02
ahy-miR164a_1ss21AG	81	26	59	3	0.33	–1.62	0.06	–4.10	4.07E-02	4.38E-05
ahy-miR166e-5p	1,350	308	1,115	401	0.23	–2.13	0.36	–1.47	1.80E-03	4.61E-03
ahy-miR166e-5p_2ss8TC10GT	1,095	200	1,197	275	0.18	–2.46	0.23	–2.12	4.00E-03	1.59E-03
ahy-miR171a	21	0	46	24	–inf	–inf	0.52	–0.95	2.64E-02	6.91E-03
ahy-miR171c-p5	71	32	142	33	0.45	–1.16	0.24	–2.08	1.33E-02	2.65E-04
ahy-miR172g_R+1_1ss9AT	7	1	9	0	0.09	–3.41	–inf	–inf	6.77E-03	3.00E-02
ahy-miR319d_L+1R-2	226	92	652	146	0.41	–1.30	0.22	–2.16	1.31E-02	1.19E-04
ahy-miR390	485	77	840	589	0.16	–2.66	0.70	–0.51	2.86E-03	4.30E-02
ahy-miR394c-p3_1ss19TC	3	0	20	6	–inf	–inf	0.27	–1.87	8.02E-03	2.93E-02
ahy-miR395g-p5	17	9	24	11	0.54	–0.88	0.47	–1.08	1.94E-02	4.48E-02
ahy-miR396-p3	196	96	520	347	0.49	–1.03	0.67	–0.58	1.87E-03	5.01E-03
ahy-miR398b	505	167	1,789	98	0.33	–1.60	0.05	–4.20	2.50E-03	3.34E-02
ahy-miR398d-p3	16	0	46	0	–inf	–inf	–inf	–inf	1.37E-02	3.52E-02
ahy-miR1511-p5	632	136	668	257	0.22	–2.21	0.38	–1.38	1.28E-03	2.48E-02
ahy-miR1515_L-1	58	24	37	18	0.42	–1.25	0.47	–1.09	1.45E-02	1.11E-02
ahy-miR3508	328	168	4,428	311	0.51	–0.97	0.07	–3.83	1.36E-02	4.88E-03

**Table 3:** miRNAs showing opposite expression patterns in peanut RILs 8106 and 8107 at 35 DAF.

miRNA ID	Normalized value mean				Fold-change	Log_2_	Fold-change	Log_2_	*p*-Value	*p*-Value	Changing pattern
	C1	T1	C2	T2	T1/C1	(T1/C1)	T2/C2	(T2/C2)	(T1 vs. C1)	(T2 vs. C2)	
ahy-miR159a-5p_2ss19TA20TA	737	225	229	267	0.30	–1.71	1.17	0.22	1.49E-04	6.21E-04	a
ahy-miR166e-5p_2ss10GT11CT	40	6	195	1,043	0.15	–2.69	5.36	2.42	1.78E-02	2.07E-02	a
ahy-miR167-3p_L-1	1,691	386	1,300	8,060	0.23	–2.13	6.20	2.63	9.14E-05	4.48E-03	a
ahy-miR171m-p5_1ss17CT	964	494	669	963	0.51	–0.97	1.44	0.53	1.70E-03	1.59E-02	a
ahy-miR172c-5p	90	46	61	344	0.51	–0.96	5.62	2.49	1.40E-03	2.54E-03	a
ahy-miR395a_L-1R+1	4	1	0	8	0.19	–2.41	inf	inf	2.95E-02	3.35E-02	a
ahy-miR395g-p5	22	5	11	26	0.25	–2.03	2.39	1.26	3.06E-03	1.87E-02	a
ahy-miR1507-5p_R-1_2ss5TA18AG	155	57	94	597	0.37	–1.44	6.36	2.67	7.61E-03	2.47E-02	a
ahy-miR2199_1ss17AG	9	0	6	226	–inf	–inf	39.76	5.31	3.07E-02	2.40E-02	a
ahy-miR3516-p5	33	5	146	3,097	0.15	–2.77	21.24	4.41	6.35E-03	1.00E-02	a
ahy-miR166a_1ss5AN	171	271	228	131	1.59	0.67	0.58	–0.80	2.21E-02	1.25E-02	b
ahy-miR166b_L+1_1ss22AC	192	298	212	129	1.55	0.63	0.61	–0.72	9.89E-03	1.20E-02	b
ahy-miR2118a-p5_2ss2AG20GT	549	1,213	185	113	2.21	1.14	0.61	–0.72	1.01E-02	1.99E-03	b
ahy-miR6300_R+4_1	20	52	34	15	2.66	1.41	0.44	–1.20	1.32E-04	1.09E-02	b
ahy-miR6300_R+4_2	20	52	34	15	2.66	1.41	0.44	–1.20	1.32E-04	1.09E-02	b
ahy-miR6300_R+4_3	20	52	34	15	2.66	1.41	0.44	–1.20	1.32E-04	1.09E-02	b

*^*a*^ miRNAs were down-regulated in RIL 8106 but were up-regulated in RIL 8107; ^*b*^ miRNAs were down-regulated in RIL 8107 but were up-regulated in RIL 8106.*

**Table 4:** miRNAs that showed differential expression in only one of the two peanut RILs at 35 DAF.

miRNAs ID	Normalized value mean				Fold-change	Log_2_	Fold change	Log_2_	*p*-Value	*p*-Value	Changing pattern
	C1	T1	C2	T2	T1/C1	(T1/C1)	T2/C2	(T2/C2)	(T1 vs. C1)	(T2 vs. C2)	
ahy-miR159b_R-1_1ss7GT	12	2	72	60	0.13	–2.94	0.84	–0.25	1.90E-02	6.66E-01	c
ahy-miR166a_L+1R-1_2	238	458	359	285	1.92	0.94	0.79	–0.33	1.83E-04	5.74E-02	c
ahy-miR166a_L+1R-1_1	238	458	359	285	1.92	0.94	0.79	–0.33	1.83E-04	5.74E-02	c
ahy-miR166a_R+2	116	189	159	143	1.62	0.70	0.90	–0.15	5.65E-03	2.15E-01	c
ahy-miR167-5p	213,076	433,359	194,733	186,348	2.03	1.02	0.96	–0.06	1.54E-02	5.60E-01	c
ahy-miR167a-p3_1ss14TC	39	0	73	66	–inf	–inf	0.90	–0.15	1.16E-02	3.89E-01	c
ahy-miR168c-3p_R+1	21	39	21	27	1.85	0.89	1.29	0.37	1.10E-04	2.09E-01	c
ahy-miR156b-p3_1ss13TC	34	83	152	344	2.46	1.30	2.27	1.18	7.60E-02	2.03E-02	d
ahy-miR160a	3,206	2,759	3,922	1,386	0.86	–0.22	0.35	–1.50	4.03E-01	1.49E-02	d
ahy-miR162a_R+1	684	824	610	740	1.21	0.27	1.21	0.28	2.87E-01	4.72E-02	d
ahy-miR164a	8,137	5,671	8,327	1,124	0.70	–0.52	0.13	–2.89	7.58E-02	1.49E-02	d
ahy-miR164a_1ss21AT	40	16	43	1	0.41	–1.28	0.03	–4.84	5.88E-02	7.78E-03	d
ahy-miR166a	42,373	62,469	58,263	44,635	1.47	0.56	0.77	–0.38	8.70E-02	2.06E-02	d
ahy-miR166a_1ss20CT	10,481	16,772	13,628	6,377	1.60	0.68	0.47	–1.10	1.03E-01	2.08E-03	d
ahy-miR166a_L+1	310	402	353	263	1.30	0.37	0.74	–0.43	5.15E-02	9.79E-03	d
ahy-miR166p_L+2R+1	32	19	22	9	0.58	–0.78	0.42	–1.24	1.88E-01	2.66E-02	d
ahy-miR168a	15,612	13,356	12,521	7,515	0.86	–0.23	0.60	–0.74	5.15E-01	2.17E-02	d
ahy-miR168a-p3	2,356	3,623	1,868	771	1.54	0.62	0.41	–1.28	1.97E-01	4.33E-03	d
ahy-miR171n	325	543	494	287	1.67	0.74	0.58	–0.78	7.74E-02	7.39E-03	d
ahy-miR171n_1ss20TC	1,192	1,449	1,742	517	1.22	0.28	0.30	–1.75	3.31E-01	9.38E-03	d
ahy-miR393a_R+1_1	6	10	4	42	1.53	0.61	10.36	3.37	2.55E-01	9.89E-04	d

*^*c*^ miRNAs were expressed predominantly in RIL 8106; ^*d*^ miRNAs were expressed predominantly in RIL 8107.*

In addition, 44 PC miRNAs from RIL 8106 and 58 PC miRNAs from RIL 8107 were differentially expressed between the 15 and 35 DAF stages (**Supplementary Tables S4-1, 4-2**). Among them, 20 miRNAs were common to the two peanut RILs (**Supplementary Figure S3B**), 24 miRNAs and 38 miRNAs were specifically expressed in RILs 8106 or 8107, respectively (**Supplementary Figure S3B**). Based on the screening criteria of the differentially expressed miRNAs, 74 miRNAs showed differential expression between the two lines (**Supplementary Table S7**). Of these, 12 miRNAs, such as PC-3p-12707_579 and PC-3p-55057_146, showed down-regulation in both lines at 35 DAF (**Supplementary Table S8-1**). Eight miRNAs were down-regulated in RIL 8106 but up-regulated in RIL 8107 (**Supplementary Table S8-2**). In addition, 54 miRNAs were expressed predominantly in only one line at the 35 DAF stage (**Supplementary Table S8-3** and **Figure 3A**).

### Validation of Differentially Expressed miRNAs by qPCR

**FIGURE 4:**
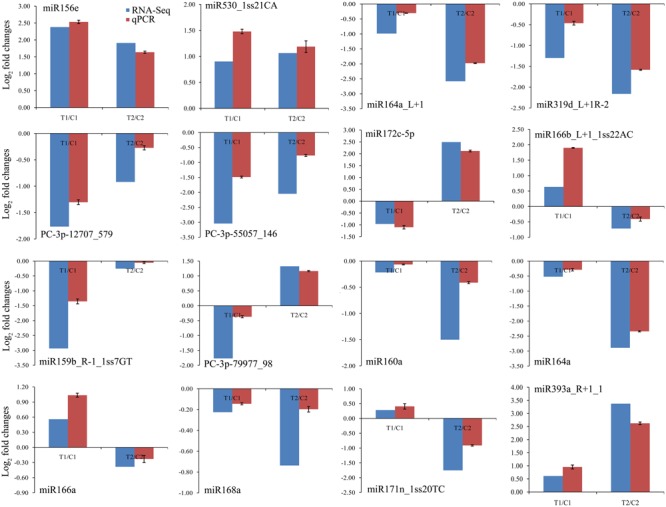
Comparisons of the expression levels of 16 miRNAs in seeds of two peanut RILs. The expression levels were normalized to the expression of *Actin* in qPCR. Red indicates the fold-changes of miRNA expression levels determined by qPCR. Blue indicates the miRNA expression fold-changes generated from the deep sequencing. The experiments were repeated three times, and vertical bars indicate the standard errors.

To confirm the high-throughput sequencing data and further comparative analyses, we verified the expression patterns of 16 randomly chosen miRNAs by qPCR. The qPCR results coincided with those of the high-throughput sequencing (**Figure 4**). For example, we confirmed that the levels of miR156e and miR530_1ss21CA were up-regulated in both peanut lines, whereas miR164a_L+1, miR319d_L+1R-2, PC-3p-12707_579, and PC-3p-55057_146 were down-regulated in both lines. Similarly, expression of miR172c-5p was shown by both methods to be down-regulated in RIL 8106 but up-regulated in RIL 8107. Conversely, miR166b_L+1_1ss22AC was shown by both methods to be down-regulated in 8107 but up-regulated in 8106. Moreover, miR160a, miR164a, miR168a, and miR171n_1ss20TC were expressed predominantly in RIL 8107. These results indicate that the frequency of occurrence as determined by high-throughput sequencing gave a reliable prediction of miRNA expression patterns.

### Identification of Target Genes of miRNAs by Degradome Analysis

Target gene validation is important to further understand the biological functions of miRNAs. In this study, we constructed two degradome libraries, DS1 (including C1 and T1) and DS2 (including C2 and T2). In total, we obtained 15,768,282 and 17,187,562 raw reads from DS1 and DS2, respectively. After removing the reads without the CAGCAG adaptor, 4,860,730 and 4,780,726 unique raw reads remained from the DS1 and DS2 libraries. The unique reads were aligned to the peanut genome database, and 4,824,026 and 4,746,614 reads were mapped to the genome, respectively. The mapped reads represented 2,916,767 and 2,979,274 annotated peanut genes in the DS1 and DS2 libraries, respectively.

**FIGURE 5:**
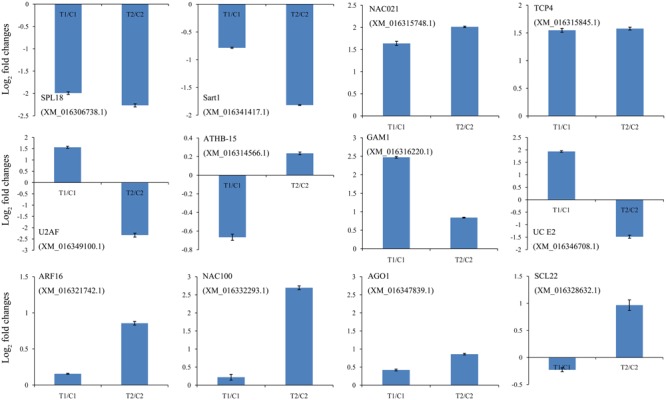
Comparison of the relative expression levels of 12 target genes in two peanut RILs. The expression levels were normalized to the expression of *Actin* in qPCR. The log_2_ fold-changes in target gene expression as determined by qPCR are shown in blue. The experiments were repeated three times and vertical bars indicate the standard errors.

We identified cleaved targets for miRNAs based on a method in the Cleaveland pipeline (Addo-Quaye et al., 2009b), in which a host gene with an alignment score of 4 or less was considered to be a potential target. In total, 1,766 and 1,616 targets were identified from the DS1 and DS2 libraries, respectively (**Supplementary Tables S9, S10**). For these targets, 501, 5, 859, 46, and 355 belonged to categories 0, 1, 2, 3, or 4 in the DS1 library, and 495, 45, 646, 40, and 390 belonged to a category <4 in the DS2 library (**Supplementary Tables S9, S10**). As expected, most of the transcripts targeted by the highly conserved miRNAs were associated with conserved target genes. For example, miR156e targets the SPL6 and SPL18 genes; miR164a_L+1 targets the NAC021 gene; miR166b_L+1_1ss22AC targets ATHB-15 gene; miR319d_L+1R-2 targets the TCP3 and TCP4 genes; miR159b_R-1_1ss7GT targets the GAM1 gene, miR160a targets the ARF10, ARF16, and ARF18 genes; miR164a targets the NAC100 gene; miR168a targets the AGO1 gene; and miR171n_1ss20TC targets the SCL22 gene. Also, the expression analysis of the 12 representative target genes by qPCR showed negative correlations with the levels of their corresponding miRNAs (**Figure 5**). These results show that several miRNAs may be directly or indirectly involved in peanut seed expansion by regulating expression of their target gene(s).

**FIGURE 6:**
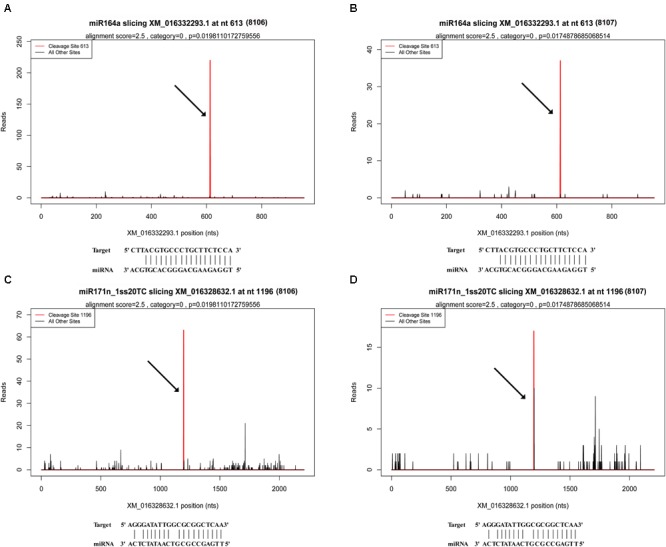
Examples of T-plots of miRNA targets in two peanut RILs confirmed by degradome sequencing. The T-plots show the distribution of the degradome tags along the full length of the target mRNA sequence. The vertical red line indicates the cleavage site of each transcript and is also shown by an arrow. **(A,B)** The cleavage features in NAC domain-containing protein 100-like (XM_016332293.1) mRNA by miR164a in the two peanut RILs, 8106 and 8107, respectively. **(C,D)** The cleavage features in scarecrow-like protein 22 (XM_016328632.1) mRNA by miR171n_1ss20TC in RILs 8106 and 8107, respectively.

There were 1,401 differentially expressed target genes between the DS1 and DS2 libraries (**Supplementary Table S11**). Examples of “target plots” (T-plots) for the targets for peanut miRNAs are shown in **Figure 6**. In this T-plot, NAC100 (XM_016332293.1) and SCL22 (XM_016328632.1) were cleaved at 613 nt and 1196 nt in the two peanut lines by miR164a and miR171n_1ss20TC, respectively (**Figure 6**). Interestingly, the read numbers of these miRNAs that cleaved the target genes in RIL 8107 showed a significant decrease compared to that in RIL 8106, which is consistent with the expression level of miRNAs identified by RNA-Seq and qPCR (**Figure 4**). Also, higher expression levels of these two targets, NAC100 and SCL22 were observed in RIL 8107 compared to the levels in RIL 8106 (**Figure 5**). The results showed that these two miRNAs may be involved in the seed expansion process in peanut by negatively regulating NAC100 and SCL22.

For the novel peanut miRNAs, 129 transcripts were identified as targets for 26 PC miRNAs in the DS1 library, and 119 transcripts were identified as targets for 23 PC miRNAs in the DS2 library (**Supplementary Tables S9, S10**). For these targets, 13, 2, 73, 5, and 36 transcripts belonged to categories 0, 1, 2, 3, and 4 in DS1, whereas 10, 3, 49, 3, and 54 transcripts belonged to categories 0, 1, 2, 3, and 4 in DS2. These data suggest that the majority of the identified PC miRNA target genes are in categories 0, 1, 2, and 3.

### Function of the Potential miRNA Targets

Gene ontology categories were assigned to 488 target genes for the 217 differentially expressed miRNAs (**Supplementary Table S12**). Twelve classes of biological processes were identified, with the two most frequent being “regulation of transcription” and “cell division and differentiation” (**Figure 3B**). Moreover, other growth and development-related genes were also identified as miRNA targets, including “auxin mediated signaling pathway” and “cell cycle” (**Figure 3B**). These results imply the possible function of miRNAs in the regulation of biological processes involved in peanut seed expansion. In addition, based on the KEGG analysis, 208 target genes were significantly enriched in 15 pathways including “plant hormone signal transduction,” “spliceosome,” and “basal transcription factors” (**Supplementary Figure S4**). Examples of the plant hormone signal transduction pathway and the corresponding miRNAs are shown in **Figure 3C**. In this pathway, miR160a targets the ARF gene to respond to the IAA signal. Another miRNA, miR171n, is involved in ubiquitin-mediated proteolysis, and controls the accumulation level of the DELLA protein in the GA pathway. These findings indicate that miRNAs have a significant effect on the regulation of peanut seed expansion by effecting hormone signaling transduction pathways.

## Discussion

### Functions of miRNAs During Peanut Seed Expansion

Peanut seed expansion, including embryogenesis, cell division, and differentiation, and seed enlargement, is a complex biological process regulated by the expression of many genes. Previous studies have indicated that miRNAs and their predicted target mRNAs play important regulatory roles in plant growth and development (Jiang et al., 2014). Nevertheless, the function of miRNAs and their targets during peanut seed expansion has not been reported. In our study, we identified 72 known miRNAs, 576 conserved miRNAs, and 434 PC miRNAs from the four libraries (**Supplementary Tables S2, S3**). Interestingly, 222 miRNAs (including miR3513-p5, miR3515-p3, and miR3516-p5) were first identified in peanut, indicating that they are preferentially expressed and are specific to peanut seed development. In addition, several PC miRNAs accumulated only in the 15 DAF or 35 DAF stages, for example, PC-5p-94368_80, PC-5p-69905_114, PC-3p-63255_126, and PC-5p-118138_60, indicating that these miRNAs may play key roles in peanut seed expansion.

### Presumptive miRNA–mRNA Modules Involved in Peanut Seed Expansion

In this study, we identified 11 up-regulated miRNAs (**Table 1**) at 35 DAF in the two peanut lines. For example, the target gene of miR156e encodes the Squamosa promoter binding protein-like (SPL) transcription factor. Previous studies have shown that SPL family members (SPL3, SPL4, and SPL5) make a secondary contribution to the regulation of flowering and appear to function mostly in the control of flowering time and developmental phase change (Wu and Poethig, 2006; Wang et al., 2009). Another study also showed that *OsSPL16* encodes a protein that promotes cell division, also with positive consequences for grain width and yield in rice (Wang et al., 2012). In this work, miR156e was up-regulated at the 35 DAF stage, while the expression pattern of the SPL gene was significantly down-regulated. The reduced expression of SPL might prolong the developmental phase associated with seed expansion.

Expression of 33 miRNAs showed down-regulation in the two peanut RILs at 35 DAF (**Table 2** and **Supplementary Table S8-1**). The target gene of miR164a_L+1encodes the NAC transcription factor, which plays a role in the early auxin response (Guo et al., 2005; Zhang et al., 2006). Another down-regulated miR319d_L+1R-2 is a member of the miR319 family and targets TCP4-like (TCP4) genes, which encode plant-specific transcription factors involved in jasmonic acid (JA) biosynthesis (Nag et al., 2009; Schommer et al., 2014). Previous studies have demonstrated that TCP positively regulates the JA level in plants (Schommer et al., 2008; Hao et al., 2012). JA has also been reported to affect grain filling in rice (Kim et al., 2009). In addition, miR319 might influence sweet corn seed vigor (Gong et al., 2015). In our present work, we found that the level of TCP4-specific mRNA increased compared with the decrease in miR319d_L+1R-2 (**Figures 4, 5**). The increased expression of TCP4 may enhance seed vigor by increasing the level of JA, which could then contribute to seed expansion in peanut.

There were 24 miRNAs that showed contrasting patterns of differential expression in the two peanut lines at 35 DAF (**Table 3** and **Supplementary Table S8-2**). For example, miR172c-5p was down-regulated in the RIL 8106, but was up-regulated in RIL 8107 (**Table 3** and **Figure 4**). The target gene of miR172c-5p is the splicing factor U2AF large subunit B-like (U2AF), which was up-regulated in RIL 8106 and down-regulated in RIL 8107 at the 35 DAF stage (**Figure 5**), indicating that U2AF may also be involved in peanut seed expansion. However, miR166b_L+1_1ss22AC was up-regulated in RIL 8106, but down-regulated in RIL 8107 (**Table 3** and **Figure 4**). miR166b_L+1_1ss22AC is a member of the miR166 family and targets the homeobox-leucine zipper protein ATHB-15 gene. In this study, we observed that expression of ATHB-15 increased with the decrease in miR166b_L+1_1ss22AC expression in RIL 8107 at 35 DAF, indicating that ATHB-15 may also play a role in peanut seed expansion by regulating early embryo development.

We also found that 95 known or conserved miRNAs (**Table 4** and **Supplementary Table S6**) and 54 PC miRNAs (**Supplementary Table S8-3**) were expressed predominantly in only one of the two peanut RILs. For example, miR159b_R-1_1ss7GT and PC-3p-79977_98 were down-regulated in RIL 8106 at 35 DAF (**Table 4** and **Supplementary Table S8-3**). miR159 negatively regulates the expression of GAMYB genes at the posttranscriptional level; GAMYB was first identified as a downstream GA signaling target in aleurone cells of barley (*Hordeum vulgare* L.) (Gubler et al., 1995). MYB family members were also found to play an important role in response to the presence of abscisic acid (ABA) during seed development in rice (Aldridge and Probert, 1993; Reyes and Chua, 2007). Our results indicate that the absence of miR159b_R-1_1ss7GT in RIL 8106 lead to increased expression of the GAMYB gene, which would be consistent with its role in peanut seed development. The target gene of PC-3p-79977_98, a novel miRNA, is the ubiquitin-conjugating enzyme E2-C (UCE2) which contributes to APC-dependent protein ubiquitylation *in vivo* during the cell cycle (Criqui et al., 2002) and cell differentiation (Wen et al., 2006). In this study, we found that UCE2 was up-regulated in concert with the down-regulation of PC-3p-79977_98 in RIL 8106 (**Figures 4, 5**), indicating that PC-3p-79977_98 may also participate in peanut seed expansion by regulating the cell cycle. Our findings suggest that the strong expression of these miRNAs in RIL 8106 could be related to a specific seed development mechanism in this line.

We also found that the expression of a number of miRNAs was down-regulated only in RIL 8107, including miR160a, miR168a, and miR171n_1ss20TC, etc. (**Table 4** and **Figure 4**). The target gene of miR160a is a member of the auxin response factor (ARF) gene family. miR160 negatively regulates ARF10 (Liu et al., 2007), ARF16 (Wang et al., 2005), and ARF17 (Mallory et al., 2005) of the repressor ARF family, and plays a critical role in maintaining the process of seed germination and the normal developmental programs for embryos, roots, leaves, and floral organs. Furthermore, ARFs regulate the expression of early auxin responsive genes, including the AUX/IAA genes (Ulmasov et al., 1997; Tiwari et al., 2003), and frees ARFs from repression by AUX/IAA proteins. The accumulation of ARF16 resulting from the down-regulation of miR160a might enhance the auxin response and thus enhance seed development in peanut. Transcription of miR168a also was significantly decreased in RIL 8107, and its target is Argonaute 1 (AGO1) which is the core component of the RNA-induced silencing complex (RISC) (Vaucheret et al., 2006) and plays crucial roles in controlling cotyledon expansion and hormone signaling (Vaucheret et al., 2004; Kidner and Martienssen, 2005). Additionally, scarecrow-like protein 22 (SCL22) was predicted to be the target gene of miR171n_1ss20TC, and it can control seed development (Kim and Ahn, 2014). We found that miR171n_1ss20TC was down-regulated in RIL 8107, indicating that SCL may also be involved in peanut seed expansion. These results suggest that genotype-specific regulation of miRNAs might explain why the seeds of RIL 8107 are larger than seeds of RIL 8106. Altogether, our results suggest that at least 10 regulatory modules of these miRNA-targets play an important role in seed expansion in peanut.

### Possible miRNA-Dependent Regulatory Pathways That Participate in Peanut Seed Expansion

Phytohormones can act as signaling compounds that promote and influence plant development. For example, auxin has been shown to be essential for plant growth and development by controlling cell division and cell elongation (Sauer et al., 2013). Gibberellic acid (GA) is involved in plant flowering and embryo development through its regulation of the expression of related genes (Blázquez and Weigel, 2000; White and Rivin, 2000). miR159 has been reported to regulate *Arabidopsis* floral development in the GA pathways; in addition, miRNA167 is involved in the auxin pathways during grain filling in rice (Achard et al., 2004; Xue et al., 2009). In this study, we also found that several miRNAs, including miR160a, miR171n, and miR156e, participate in auxin signal transduction, GA signal transduction, and BR signal transduction by regulating their target genes (**Figure 3C**). These results suggest that these miRNAs may play key roles in peanut seed expansion.

**FIGURE 7:**
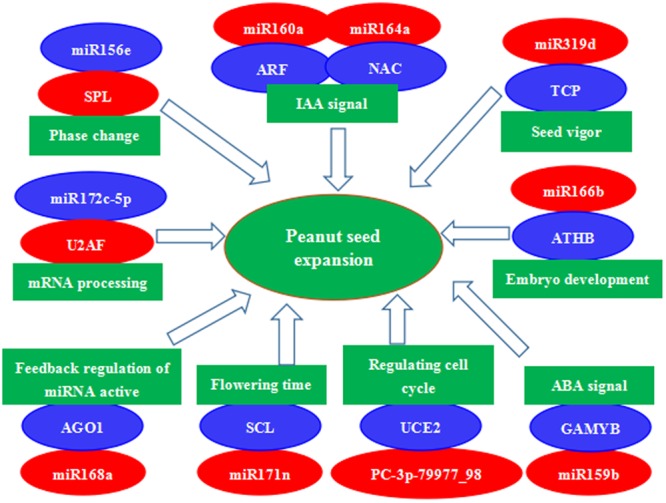
A proposed regulatory mechanism involving differentially expressed miRNAs and their target genes during peanut seed expansion. Red indicates down-regulation and blue indicates up-regulation of gene expression.

A number of other conserved and novel miRNAs may also be involved in peanut seed expansion, and we further assessed the functions of their potential target genes in seed development. Examples are miR164a-mediated cleavage of NAC, miR319d-mediated cleavage of TCP, miR166b-mediated cleavage of ATHB, miR159b-mediated cleavage of GAMYB, PC-3p-79977_98-mediated cleavage of UCE2, miR168a-mediated cleavage of AGO1, and miR172c-5p-mediated cleavage of U2AF. These miRNA–mRNA modules might be involved in regulating seed expansion in peanut (**Figure 7**). In summary, miRNA-target gene models, hormone transduction, and transcriptional regulation comprise a complex network that regulates seed expansion in peanut.

## Conclusion

In this study, we found that 143 conserved miRNAs and 74 PC miRNAs were differentially expressed in the two peanut RILs at the 35 DAF stage. Of these, expression of 11 miRNAs was found to be up-regulated, while 33 miRNAs were down-regulated. Twenty-four miRNAs displayed contrasting expression patterns in the two peanut lines. Moreover, 149 miRNAs were expressed predominantly in only one of the two RILs. We also validated the expression of a number of representative miRNAs by qPCR. The potential miRNA target genes were verified by sequencing the two degradome libraries. Our results demonstrate that the expression patterns of some miRNAs can be very different even between two closely related inbred lines from the same species. The differentially expressed miRNAs and their target genes identified in this study could be important to the regulatory networks that control seed expansion in peanut.

## Author Contributions

DY designed and conceived the research. XM and ZX wrote the manuscript. XM and XZ analyzed the data. KZ, FL, KL, and LN performed the experiments. ZX, JH, and DY edited the manuscript. All authors read and approved the final manuscript.

## Conflict of Interest Statement

The authors declare that the research was conducted in the absence of any commercial or financial relationships that could be construed as a potential conflict of interest.

## Footnotes

1 http://www.ncbi.nlm.nih.gov/genbank/

2 http://www.girinst.org/repbase/update/index.html

3 http://www.sanger.ac.uk/software/Rfam

4 http://www.mirbase.org

5 ftp://ftp.ncbi.nlm.nih.gov/genomes/all/GCA/000/816/755/GCA_000816755.1_Araip1.0/

6 ftp://ftp.ncbi.nlm.nih.gov/genomes/all/GCA/000/817/695/GCA_000817695.1_Aradu1.0/

7 http://sourceforge.net/projects/mireap/

8 http://rna.tbi.univie.ac.at/cgi-bin/RNAWebSuite/RNAfold.cgi

9 http://compgen.bio.unipd.it/bioinfo/software/

10 http://www.geneontology.org/

11 http://www.ncbi.nlm.nih.gov/

12 http://www.uniprot.org/

13 http://www.genome.jp/kegg/

14 http://apps.cytoscape.org/apps/cluego

15 http://www.cytoscape.org/

## Supplementary Material

FIGURE S1 Size distribution of peanut small RNAs. C1 and C2 are small RNAs isolated from RILs 8106 and 8107 at 15 DAF, respectively. T1 and T2 are the small RNAs isolated from RILs 8106 and 8107 at 35 DAF, respectively.

FIGURE S2 Common and specific miRNAs in four small RNA libraries prepared from developing peanut seeds. Venn diagrams showing the number of known miRNAs **(A)**, conserved miRNAs **(B)**, and PC miRNAs **(C)**.

FIGURE S3 Common differentially expressed miRNAs and specifically expressed miRNAs in the two peanut RILs at 35 DAF. Venn diagrams showing the number of known miRNAs and conserved miRNAs **(A)** and PC miRNAs **(B)**.

FIGURE S4 KEGG analysis of the predicted target genes of the differentially expressed miRNAs isolated from developing peanut seeds.

TABLE S1 Annotation of sRNAs sequences from C1, T1, C2, and T2.

TABLE S2 All expressed known and conserved miRNA.

TABLE S3 All predicted candidate miRNAs (PC miRNAs) in this study.

TABLE S4 Differentially expressed miRNAs in four comparisons. **(S4-1)** Differentially expressed miRNAs in 8106 at 35 DAF (T1vs. C1). **(S4-2)** Differentially expressed miRNAs in 8107 at 35 DAF (T2 vs. C2). **(S4-3)** Differentially expressed miRNAs in C2 vs. C1. **(S4-4)** Differentially expressed miRNAs in T2 vs. T1.

TABLE S5 All differentially expressed miRNAs between two peanut lines at 35 DAF.

TABLE S6 95 differentially expressed miRNAs only in one peanut line at 35 DAF.

TABLE S7 All differentially expressed PC miRNAs between two peanut lines at 35 DAF.

TABLE S8 Differentially expressed PC miRNAs in two peanut lines at 35 DAF. **(S8-1)** Down-regulated PC miRNAs in two peanut lines at 35 DAF. **(S8-2)** Opposite expression PC miRNAs in two peanut lines at 35 DAF. **(S8-3)** Differentially expressed miRNAs only in one peanut line at 35 DAF.

TABLE S9 All targets of miRNAs in DS1.

TABLE S10 All targets of miRNAs in DS2.

TABLE S11 1401 differentially expressed target genes between the DS1 and DS2 libraries.

TABLE S12 Potential target genes of differentially expressed miRNAs.

TABLE S13 All primer sequences in this study. **(S13-1)** Primer sequences used for qPCR analysis of 16 differentially expressed miRNAs. **(S13-2)** Primer pair sequences used for qPCR analysis of 12 target genes.
