# Supplementary figures and images for: Small RNA and Degradome Deep Sequencing Reveals the Roles of microRNAs in Seed Expansion in Peanut (Arachis hypogaea L.)
Source: Front Plant Sci. 2018 Mar 20;9:349. doi: 10.3389/fpls.2018.00349 (PMC5890158; doi:10.3389/fpls.2018.00349)

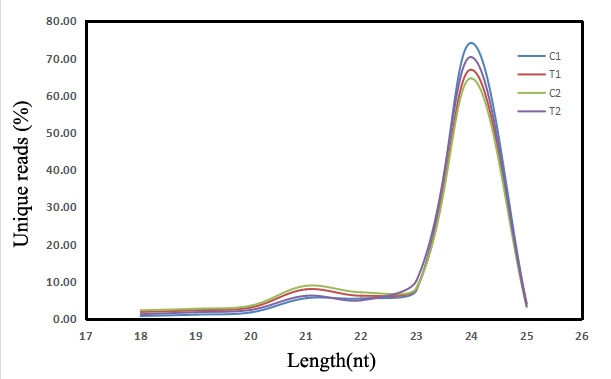

Supplement: FIGURE S1 — Size distribution of peanut small RNAs. C1 and C2 are small RNAs isolated from RILs 8106 and 8107 at 15 DAF, respectively. T1 and T2 are the small RNAs isolated from RILs 8106 and 8107 at 35 DAF, respectively. [file Image_1.TIF]

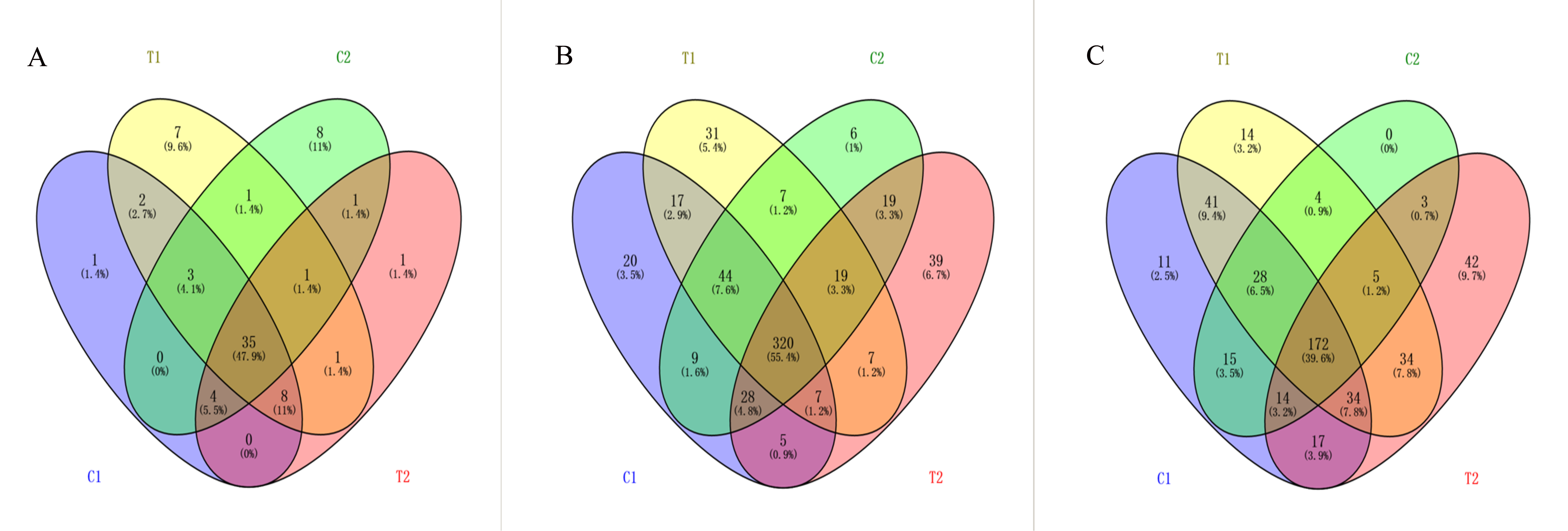

Supplement: FIGURE S2 — Common and specific miRNAs in four small RNA libraries prepared from developing peanut seeds. Venn diagrams showing the number of known miRNAs (A), conserved miRNAs (B), and PC miRNAs (C). [file Image_2.TIF]

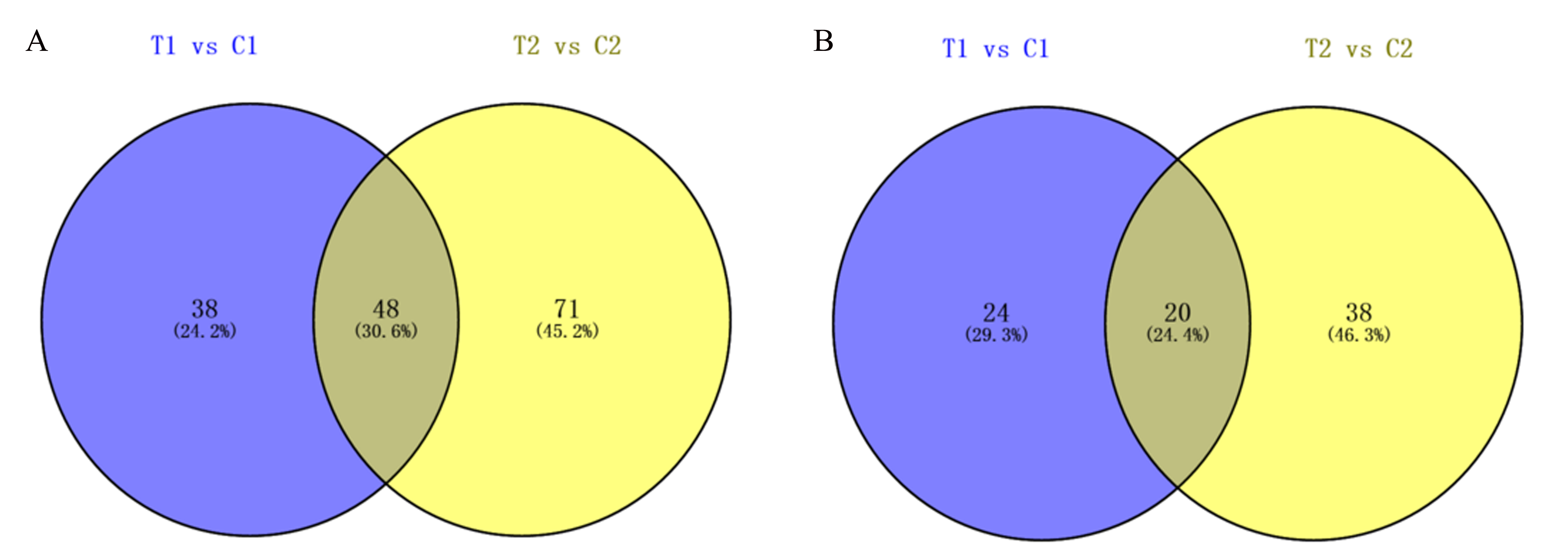

Supplement: FIGURE S3 — Common differentially expressed miRNAs and specifically expressed miRNAs in the two peanut RILs at 35 DAF. Venn diagrams showing the number of known miRNAs and conserved miRNAs (A) and PC miRNAs (B). [file Image_3.TIF]

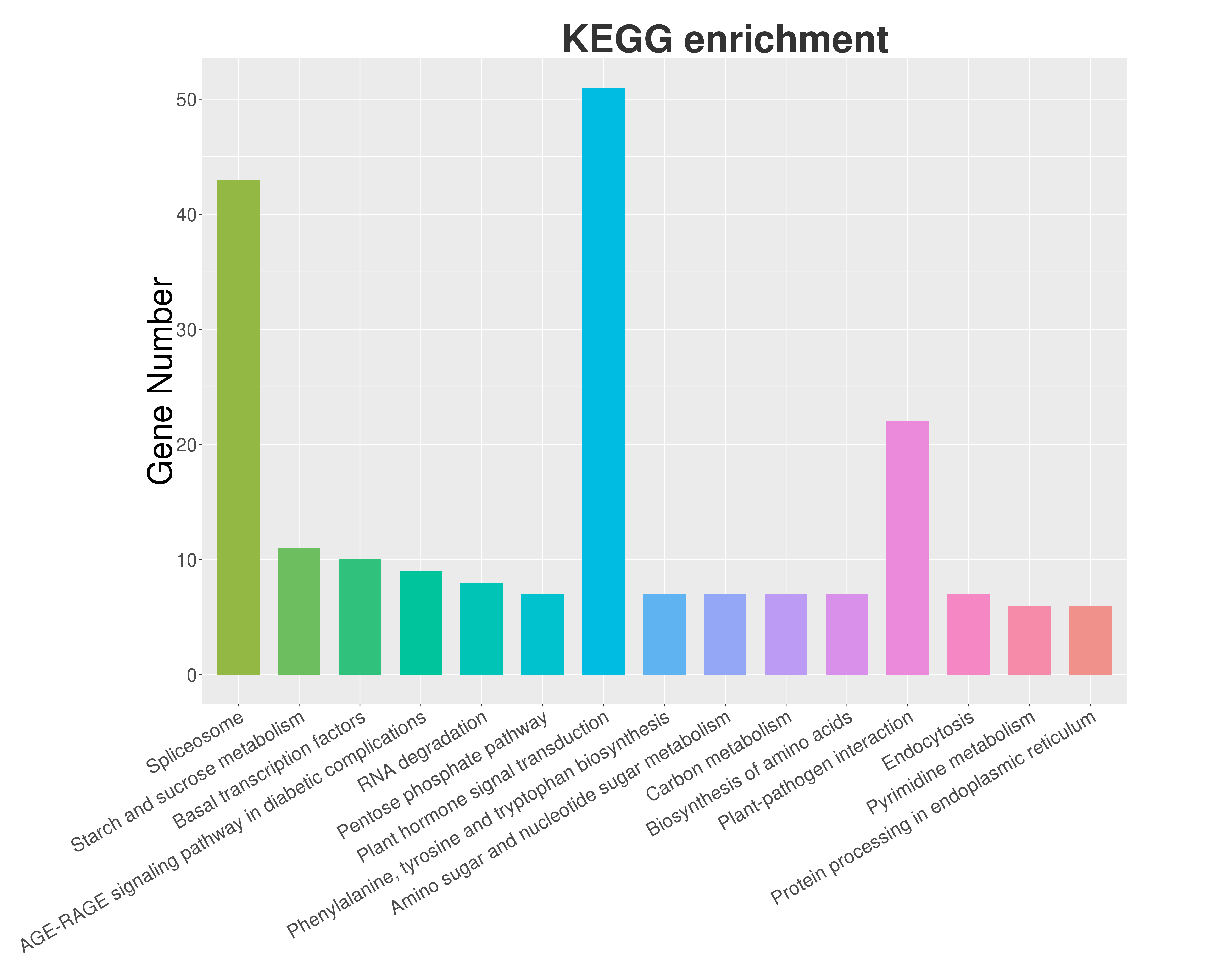

Supplement: FIGURE S4 — KEGG analysis of the predicted target genes of the differentially expressed miRNAs isolated from developing peanut seeds. [file Image_4.TIF]
